# Biosurfactant from Nile Papyrus endophyte with potential antibiofilm activity against global clones of *Acinetobacter baumannii*

**DOI:** 10.3389/fcimb.2023.1210195

**Published:** 2023-07-13

**Authors:** Mai A. Amer, Reham Wasfi, Samira M. Hamed

**Affiliations:** Department of Microbiology and Immunology, Faculty of Pharmacy, October University for Modern Sciences and Arts (MSA), Giza, Egypt

**Keywords:** *Acinetobacter baumannii*, global clones, endophytes, central venous catheter (CVC), biosurfactant, antibiofilm, *Bacillus amyloliquefaciens*, Papyrus

## Abstract

*Acinetobacter baumannii* is a leading cause of biofilm-associated infections, particularly catheter-related bloodstream infections (CRBSIs) that are mostly recalcitrant to antimicrobial therapy. One approach to reducing the burden of CRBSIs is inhibiting biofilm formation on catheters. Owing to their prodigious microbial diversity, bacterial endophytes might be a valuable source of biosurfactants, which are known for their great capacity to disperse microbial biofilms. With this in mind, our study aimed to screen bacterial endophytes from plants growing on the banks of the River Nile for the production of powerful biosurfactants capable of reducing the ability of *A. baumannii* to form biofilms on central venous catheters (CVCs). This was tested on multidrug- and extensive drug-resistant (M/XDR) clinical isolates of *A. baumannii* that belong to high-risk global clones and on a standard strain of *A. baumannii* ATCC 19606. The drop collapse and oil dispersion assays were employed in screening the cell-free supernatants (CFS) of all endophytes for biosurfactant activity. Of the 44 bacterial endophytes recovered from 10 plants, the CFS of *Bacillus amyloliquefaciens* Cp24, isolated from *Cyperus papyrus*, showed the highest biosurfactant activity. The crude biosurfactant extract of Cp24 showed potent antibacterial activity with minimum inhibitory concentrations (MICs) ranging from 0.78 to 1.56 mg/ml. It also showed significant antibiofilm activity (p-value<0.01). Sub-MICs of the extract could reduce biofilm formation by up to 89.59%, while up to 87.3% of the preformed biofilms were eradicated by the MIC. A significant reduction in biofilm formation on CVCs impregnated with sub-MIC of the extract was demonstrated by CV assay and further confirmed by scanning electron microscopy. This was associated with three log_10_ reductions in adhered bacteria in the viable count assay. GC-MS analysis of the crude biosurfactant extract revealed the presence of several compounds, such as saturated, unsaturated, and epoxy fatty acids, cyclopeptides, and 3-Benzyl-hexahydro-pyrrolo [1, 2-a] pyrazine-1,4-dione, potentially implicated in the potent biosurfactant and antibiofilm activities. In the present study, we report the isolation of a *B. amyloliquefaciens* endophyte from the plant *C. papyrus* that produces a biosurfactant with potent antibiofilm activity against MDR/XDR global clones of *A. baumannii*. The impregnation of CVCs with the biosurfactant was demonstrated to reduce biofilms and, hence, proposed as a potential strategy for reducing CRBSIs.

## 1 Introduction

*Acinetobacter baumannii* (*A. baumannii*) has become a global threat in healthcare settings and a leading cause of healthcare-associated infections (Gedefie et al., 2021). This successful nosocomial pathogen is known for its adaptable genetic machinery that is capable to accumulate resistance genes and to acquire multidrug-, extensive drug-, and pan-drug-resistance phenotypes (Rolain et al., 2013; Wasfi et al., 2021; Zafer et al., 2021). In addition, it has remarkable environmental resilience partly due to its simple growth requirements and resistance to desiccation (Peleg et al., 2012).

One of the hallmark characteristics of *A. baumannii* is the propensity to form biofilms in which they live in surface-attached communities (Eze et al., 2018). Biofilm-embedded *A. baumannii* is properly shielded from antibiotics, immunity factors, and harsh environmental conditions. The close proximity within biofilms further enhances the acquisition of foreign genes through horizontal gene transfer. Hence, *A. baumannii* is a frequent cause of biofilm-related, particularly catheter-related bloodstream infections (CRBSI) and ventilator-associated pneumonia (VAP) (Gedefie et al., 2021). Such infections are mostly associated with devastating outcomes and are exceedingly resistant to a wide range of antimicrobial treatment modalities, posing a great challenge to infectious disease practitioners (Mansouri et al., 2013; Abd El-Rahman et al., 2023; Lafuente Cabrero et al., 2023).

Biofilm-related *A. baumannii* infections are mostly seen in intensive care units (ICU), where life support systems (e.g.; mechanical ventilation) and indwelling medical devices (e.g.; vascular and urinary catheters) are widely used (Lynch et al., 2017). A central venous catheter (CVC) insertion is the most common invasive procedure that leads to *A. baumannii* infections in ICUs (Castilho et al., 2017). Even using extreme aseptic techniques, the percutaneous insertion of catheters was found to permit the attachment of bacteria. This then progresses to biofilm formation and planktonic dispersion into the bloodstream, causing CRBSIs (Pathak et al., 2018). An estimated 250,000 bloodstream infections occur annually, and most are related to the presence of intravascular devices. In the United States, CRBSIs are still experienced by tens of thousands of patients annually, resulting in thousands of deaths and adding billions of dollars to the cost of the country’s healthcare system (Guenezan et al., 2018). The likelihood of developing CRBSI was found to be increased by chemotherapy, immunosuppression, and long-term catheterization (Lafuente Cabrero et al., 2023). Previous studies have reported biofilm formation capacity in up to 76% of CVC devices reported to have *A. baumannii* (Nahar et al., 2013; Sanchez et al., 2013; Duarte et al., 2016; Castilho et al., 2017). Consequently, researchers have been long looking for innovative ways to maintain CVCs biofilm-free.

Catheter coating or impregnation with various compounds that prevent biofilm formation and bacterial colonization is one of the approaches proposed by many authors to reduce the incidence of CRBSIs. While antimicrobial agents have been commonly used for coating or impregnation of catheters (Wang et al., 2018; Neethu et al., 2020; Sivanandan, 2020; Corrêa Carvalho et al., 2022), only partial clinical efficacy was shown by some antimicrobial-coated catheters, particularly against MDR pathogens (Mansouri et al., 2013). In addition, the use of antimicrobial-treated catheters might contribute to the evolution of antimicrobial resistance (Donlan, 2011). Hence, antibiofilm compounds represent good alternatives to antimicrobial agents, as they inhibit biofilms without exerting selection pressure on bacterial growth and thus reduce the development of antibiotic resistance (Amer et al., 2022, Wang et al., 2018; Neethu et al., 2020). Examples include catheter pretreatment with bacteriophages, surfactants, or enzymes (Donlan, 2011).

Biosurfactants are among the promising candidates for application in inhibiting bacterial biofilms (Banat et al., 2014). Biosurfactants, also named green surfactants, are surface active agents of biological origin. They are amphiphilic in nature, having hydrophilic and hydrophobic parts. They are non-toxic and biodegradable and do not accumulate in the environment. Microbial biosurfactants have been gaining much attention, owing to their chemical properties and stability under several environmental conditions (Eras-Muñoz et al., 2022). These properties make them relevant molecules for applications in combating many diseases and as potential therapeutic agents.

Endophytes are a class of endosymbiotic microorganisms that colonize plants and serve as stores for unique bioactive secondary metabolites, such as alkaloids, phenolic acids, quinones, steroids, saponins, tannins, terpenoid, and biosurfactants (Gouda et al., 2016; Ashitha et al., 2020; Marchut-Mikołajczyk et al., 2021). Endophytes can stimulate plant growth, facilitate the *de novo* synthesis of biologically active compounds, such as antibiotics, biosurfactants, and phytohormones, increase the host’s resistance to stressful environmental conditions, and increase the resistance of the host plant to pathogens and pests (Marchut-Mikołajczyk et al., 2021). Research into the biodiversity of endophytic strains for novel metabolites may lead to the discovery of new drugs, potentially contributing to the effective treatment of diseases in humans, plants, and animals (Ryan et al., 2008). Ongoing discoveries on the variety of metabolites produced by endophytes and their promising applications show that endophytes have inspired research in the development of biotechnological solutions. These solutions span from the exploration to the manufacture of industrially relevant metabolites that could help identify long-lasting sustainable solutions for the economic exploitation of biosurfactants that reduce biofilm formation, (Tidke et al., 2019).

Thus, the present study aimed to screen endophytes from Egypt for the ability to produce a powerful biosurfactant that can inhibit biofilms of MDR and XDR *A. baumannii* and to investigate the potential application of this biosurfactant in reducing biofilm formation on CVCs.

## 2 Materials and methods

### 2.1 Clinical strains and growth conditions

Five clinical isolates of multidrug- and extensive drug-resistant *A. baumannii* and a standard strain *A. baumannii* ATCC^®^ 19606 were included in this study. The clinical isolates were recovered from different clinical specimens of patients admitted to Kasr Al-Ainy Hospital, which were collected in a previous study conducted by Hamed et al. (2022). As part of the previous study, the multilocus sequence typing (MLST) revealed that these isolates belong to high-risk global clones (GCs), as shown in **Table 1**. Bacterial cultures were routinely grown in Luria-Bertani (LB) medium at 37°C for 24 hours.

**Table 1:** MLSTs and GCs of the clinical isolates included in the current study (Hamed et al., 2022).

Isolate number	Specimen	Resistance Phenotype	ST^Pas^	ST^Oxf^	GC
M02	Wound swab	XDR	85	1089	9
M03	Blood	XDR	113	2246	7
M04	Sputum	XDR	2	1816/195	2
M15	Wound swab	MDR	1	1604/231	1

XDR, extensive drug resistance; MDR, multidrug resistance; ST^Pas^, sequence type based on Pasteur scheme; ST^Oxf^, sequence type according to Oxford scheme; GC, global clone.

### 2.2 Collection of plants and isolation of endophytes

From April to November 2019, samples from different plants growing along the banks of the River Nile were randomly collected and screened for biosurfactant-producing bacterial endophytes. For endophyte isolation, fresh and healthy samples of each plant were collected, stored in sealed plastic bags, and delivered to the laboratory on the same day of collection for further processing. All samples were surface sterilized in the method described by Katoch et al. (2017), with some modifications. Briefly, the samples were thoroughly washed under tap water and distilled water, then dried using tissue paper. All washed plant materials were cut into 5 cm segments that were surface sterilized by sequential immersion in 70% ethanol (v/v) for 3 min, 1% sodium hypochlorite (v/v) for 12 minutes, and then 70% ethanol (v/v) for additional 30 sec. Finally, the segments were rinsed three times in sterile distilled water to remove residual sterilant and then left to dry under an airflow cabinet until complete drying. The plant segments were aseptically cut using sterile surgical scalpels into 2 mm-thick segments that were placed on trypticase soy agar (TSA) plates, ensuring direct contact of the cut edges with the culture media. The plates were incubated at 30°C for 5 days. To confirm the effectiveness of the surface sterilization process, 50 µl samples of the last distilled water rinse were cultured on TSA, incubated at the same conditions, and checked daily for the growth of surface contaminants. Morphologically distinct bacterial colonies growing under the plant parts were isolated to obtain pure colonies. Endophyte isolates were preserved in trypticase soy broth (TSB) supplemented with 25% (v/v) glycerol at -20°C.

### 2.3 Screening the endophytes for biosurfactant production

Endophytes were screened for biosurfactant production after the preparation of cell-free supernatant (CFS) using the methods described by Amer et al. (2021). The turbidity of an overnight culture of endophytes grown in TSB was adjusted to be equivalent to an OD_600_ of 1. The diluted culture was then used to inoculate fresh TSB to achieve a final dilution of 1:100. Flasks were then incubated at 30°C with shaking at 120 rpm for 4 days. CFS was prepared by centrifugation at 10,000 rpm for 10 min at 4°C, followed by filtration using a 0.22 µm filter (Millipore, Bedford, MA, USA). These were then screened for their biosurfactant activity using the drop collapse, oil displacement, and emulsification assays described below.

#### 2.3.1 Drop collapse assay

The drop collapse assay was carried out as described by Patel et al. (2021). First, a drop (35 µl) of the CFS was placed on the surface of a parafilm. Biosurfactant production was indicated by the spreading or collapse of the drop within 15 min (Patel et al., 2021).

#### 2.3.2 Oil displacement test

Isolates that showed positive drop collapse assay were further subjected to the oil displacement assay that was carried out following the procedure described by Joe et al. (2019). Briefly, 50 ml of distilled water was added to a Petri dish. This was overlaid by a thin layer of castor oil (100 µl). Approximately 10 µl of the CFS was added to the center of the oil layer. After 30 seconds, the oil surface was observed for the emergence of a clear zone. Uninoculated TSB and Triton X-100 were used as negative and positive controls, respectively.

The isolate with CFS causing the greatest displacement of the oil layer was selected for further testing.

#### 2.3.3 Emulsification assay

The emulsification assay confirmed the biosurfactant production potential of the endophyte that showed the highest oil displacement activity. Following the method described by Satpute et al. (2010), equal parts of the CFS and castor oil were combined by vortexing for 2 minutes before being left to stand for 24 hours. The emulsification activity was indicated by the emulsification index (% EI24), which was calculated according to the following formula:


%EI24=(Height of the formed emulsion/Total height of the solution)×100

### 2.4 Molecular identification of the bacterial endophyte-producing biosurfactant

The bacterial endophyte showing the highest emulsification activity was identified by its microscopic morphology, followed by molecular analysis based on the 16S *rRNA* gene sequence. DNA was extracted using the GeneJET Genomic DNA Purification Kit (Thermo Fisher Scientific Inc., USA) as per the manufacturer’s instructions. The 16S *rRNA* gene was amplified by the universal pair of primers designed by Weisburg et al. (1991): 27F (5′AGAGTTTGATCCTGGCTCAG3′) and 1492R (5′CGGTTACCTTGTTACGACTT3′). The purified PCR product was then sequenced by Macrogen^®^ (Seoul, South Korea) using ABI 3730xl DNA Analyzer. Gene sequences were compared to sequences in the National Center for Biotechnology Information (NCBI) database using the nucleotide Basic Local Alignment Search Tool (BLASTn). Gene sequences with high similarity to that determined in the study were retrieved and genetic diversity was analyzed using Molecular Evolutionary Genetics Analysis version 11.0 (MEGA 11). The phylogenetic tree was constructed by the maximum parsimony method (Tamura et al., 2013).

### 2.5 Preparation of the crude biosurfactant from the endophyte Cp24

For extraction of the crude biosurfactant, the CFS was prepared in the same way as in the preliminary screening for biosurfactant production. The CFS was acidified to pH 2.5 using HCl (5 N) and stored overnight at 4°C for precipitation of the biosurfactant compounds. The crude biosurfactant was then extracted twice from the acidified supernatant by shaking with double volumes of ethyl acetate (EtOAc) (Patel et al., 2021). The ethyl acetate extract was pooled and dried under a vacuum in a rotary flash evaporator (Heidolph, Germany) at 45°C. Based on the solubility, the dried extract was dissolved in 20% (v/v) Dimethylsulfoxide (DMSO) to obtain a stock solution of 50 mg/ml. The final concentrations of DMSO in all experiments were confirmed to not affect bacterial growth.

### 2.6 Assessment of the antibacterial activity of the crude biosurfactant against *A. baumannii*

The antibacterial activity of the crude biosurfactant extract against *A. baumannii* was evaluated using the broth microdilution assay. The minimum inhibitory concentrations (MICs) of the crude biosurfactant were determined according to the guidelines of the Clinical and Laboratory Standards Institute (CLSI) (CLSI, 2015). In 96-well microtiter plates, two-fold serial dilutions of the crude extract were prepared in Muller Hinton Broth (MHB; Oxoid, UK) to final concentrations ranging from 0.01 to 12.5 mg/ml. All wells were inoculated with approximately 5×10^5^ CFU ml^−1^ of each test strain. After overnight incubation at 37°C, the MICs were visually recorded. Negative controls were prepared with MHB containing DMSO at the same concentrations as the extract.

### 2.7 Assessment of the antibiofilm activity of the crude biosurfactant against *A. baumannii*

#### 2.7.1 Effect of the crude biosurfactant on bacterial adherence and biofilm formation

The ability of clinical strains to form biofilm was assayed using a crystal violet stain as described by Amer et al. (2021). Briefly, overnight cultures of *A. baumannii* strains adjusted to a count of 1.5 x 10^8^ CFU/ml were diluted 1:50 in LB broth supplemented by 1% (w/v) glucose. Then 200 µl of the dilute cultures were inoculated into a 96-well flat-bottomed polystyrene microtiter plate (Greiner Bio One, Germany) The plates were incubated in static conditions for 24 h at 37°C. Following incubation, the planktonic microbial growth was then measured at a wavelength of 600 nm (OD_growth_Planktonic) using an ELISA plate reader (Stat Fax^®^2100) Awareness Technology (Palm City, FA, USA). The wells were then washed three times with phosphate-buffered saline (PBS, pH=7.4) to remove unadhered or loosely adhered cells. After air drying, biofilms were stained with 0.1% (w/v) crystal violet (CV) solution for 15 min. The plates were then washed with water to rinse off the excess stain. The CV stain bound to the adherent cells was then solubilized by 33% glacial acetic acid and the biofilm biomass was quantified colorimetrically (OD_CV_ Biofilm) at 570 nm. To reduce background signals, a blank containing an uninoculated medium was included and measured (OD_growth_ Blank and OD_CV_ Blank). The biofilm index (BFI) of each clinical strain was calculated using the following equation:


$$\text{BFI }=\;(\text{O}{\text{D}}_{\text{CV}}\text{ Biofilm}-\text{O}{\text{D}}_{\text{CV}}\text{ Blank})/(\text{O}{\text{D}}_{\text{growth}}\text{ Planktonic}-\text{O}{\text{D}}_{\text{growth}}\text{ Blank})$$

Isolates were classified into non-adherent, weak, moderate, and strong biofilm-forming isolates according to the semiquantitative classification of biofilm production as described by Naves et al. (2008). The effect of the crude biosurfactant on biofilm formation by *A. baumannii* was tested as described by Amer et al. (2021). A volume of 100 µl of the diluted culture was inoculated into a 96-well flat-bottomed polystyrene microtiter plate (Greiner Bio-one^®^, Germany) containing equal volumes of the crude biosurfactant at concentrations equivalent to the MIC, reaching a final concentration of 0.5X MIC. Negative controls containing DMSO at the same final concentrations as in the crude biosurfactant (control) were also included. Biofilm was stained by crystal violet and BFI was determined. The antibiofilm activity of the crude biosurfactant was expressed as percentage biofilm inhibition (%BI) that was calculated according to the following formula:


%BI = [(BFI (control)− BFI (test))/BFI (control)] ×100

Where BFI (test) and BFI (control) are the BFIs of each strain in the presence and absence of the crude biosurfactant, respectively.

#### 2.7.2 Effect of the crude biosurfactant on established biofilms

The efficacy of the crude biosurfactant in eradicating established biofilms was assessed using the method, with slight modifications, described by Lemos et al. (2018). First, 100 µl of overnight cultures of *A. baumannii* adjusted to 10^6^ cells/ml in LBG were transferred to 96-well microtiter plates to form biofilms (Lemos et al., 2018). After overnight incubation in static conditions at 37°C, planktonic cells were delicately removed, and the wells were washed three times with PBS. The adherent cells remaining in the wells were then treated with 200 µl of the crude biosurfactant at concentrations equivalent to MIC. Wells treated with DMSO at the same final concentrations as in the crude biosurfactant served as control. The plates were incubated at 37°C for an additional 24 h, after which the supernatants were removed, and wells were washed three times using PBS. The residual biofilms were quantified using two methods, namely, crystal violet staining as described in section 2.7.1 and viable count assays as described by Ziemyte et al. (2020). Using the crystal violet staining method, the biofilm eradication percentage was calculated using the following formula (Patel et al, 2021):


$$\text{Biofilm eradication percentage}=\left[(\text{O}{\text{D}}_{\text{CV}}\text{ Control }-\text{O}{\text{D}}_{\text{CV}}\text{ Test})/\text{O}{\text{D}}_{\text{CV}}\text{ Control}\right]\times 100$$

Where OD_CV_ Control is the absorbance reading of control; OD_CV_ Test is the absorbance reading of biosurfactant treated biofilm.

To determine the number of viable biofilm-embedded bacteria after treatment with the crude biosurfactant, the wells were filled with 200 µl PBS and adherent cells were detached by sonication for 5 min. The viable count was determined using the drop plate method as described by Herigstad et al. (2001). Tenfold serial dilutions were prepared and 10 µl were plated onto MacConkey agar plates in triplicates, and incubated at 37°C for 24 h. The biofilm eradication percentage was expressed as a log_10_ reduction of the viable count in the biofilms treated by the crude biosurfactant compared to the negative control (Amer et al., 2022).

### 2.8 Evaluation of the antibiofilm effect of crude biosurfactant on central venous catheters (CVCs) *in vitro*

The catheter model was performed with a triple-lumen polyurethane CVC (Amecath^®^ Ref. No. CTLC-0720-KGSN, Ameco Medical Industries, Egypt). In this model, the biosurfactant-impregnated CVC was challenged by the *A. baumannii* strain (M02) that showed the highest capacity for biofilm formation.

#### 2.8.1 Preparation of the biosurfactant-impregnated CVC

The CVC was divided into 1-cm-long segments. Each was impregnated with the crude biosurfactant at a final concentration of 0.5X MIC (0.78 mg/ml) and kept at room temperature for 24 h (Amer et al., 2022). The catheters were then air-dried to restore their original size. Control segments were impregnated with DMSO at the same concentration used in the test.

#### 2.8.2 Antibiofilm assay

The inhibitory effect of the crude biosurfactant on the ability of *A. baumannii* M02 (strong biofilm-forming isolate) to form biofilm on the CVC was evaluated according to the method, with slight modifications, described by Raad et al. (2012). First, the biosurfactant-impregnated and the control segments of the CVC were conditioned by plasma from a human volunteer (one of the authors). For this purpose, all CVC segments were placed in a sterile 12-well culture plate containing 2 ml of human plasma and incubated for 24 h at 37°C. The plasma was then replaced by LBG inoculated by ~1X10^6^ CFU/ml of the test strain and the plate was incubated at 37°C for an additional 24 h. After incubation, the catheter sections were gently washed with sterile PBS to remove the non-adhered planktonic cells. The biofilms formed on the control and the biosurfactant-impregnated CVC segments were quantified by crystal violet staining and the viable count assay as described by Amer et al. (2022). For a detachment of the biofilm-embedded cells to determine the remaining viable bacteria on the catheter surface, CVC segments were sonicated in 1 ml PBS for 15 min, followed by 5 min vortexing. Aliquots of PBS were used for viable bacterial counting.

#### 2.8.3 Scanning electron microscope analysis of biofilms on CVC

The efficacy of the CVC biosurfactant-impregnation against *A. baumannii* M02 biofilm was further confirmed through scanning electron microscope (SEM) analysis. Biofilms of *A. baumannii* M02 were allowed to develop on the biosurfactant-impregnated and the control CVC segments, as described in the antibiofilm assay. The segments were washed with PBS and then fixed using 2.5% glutaraldehyde. This was followed by gradual dehydration using increasing concentrations of 20, 40, 60, 80, and 100% ethanol. The dehydrated samples were plated by gold sputter for examination under SEM (Quanta™ 250 FEG, Thermo Fischer Scientific; New Hampshire, USA) (Yassin et al., 2019).

### 2.9 Cytotoxicity assay of the biosurfactant crude extract

The cytotoxicity assay of the crude biosurfactant extract was adapted from the ISO 10993-5 protocol. The assay was carried out using healthy human skin fibroblast cells obtained from Nawah Scientific Inc. (ATCC CCL-75) (Hou et al., 2020; Ivanova et al., 2021).

The biosurfactant crude extract was embedded in serum-free Dulbecco’s Modified Eagle Medium (DMEM) overnight at 37°C. At the same time, the human fibroblast cell line was cultured in DMEM supplemented with 10% v/v fetal bovine serum (FBS) and 1% w/v penicillin−streptomycin for 24 h at 37°C, 5% v/v CO2.

After achieving the confluence, 100 µl of the cell suspension (5X10^3^ cells/ml) was seeded in each well of the 96-well plate. Then, the cells were incubated overnight in a humidified atmosphere (>90% humidity) with 5% CO2 at 37°C. Cells were treated with another aliquot of 100 μl media containing biosurfactant extract at various concentrations. After 72 h of treatment, cells were fixed by replacing media with 150 μl of 10% trichloroacetic acid (TCA) and incubated at 4°C for 1 h. The TCA solution was removed, and the cells were washed 5 times with PBS. The viability of the cells was evaluated using sulforhodamine B (SRB) (0.4% w/v) assay as described by Allam et al. (2018), and color intensity was measured at wavelength 540 nm. The percentage of cell viability was calculated using the following formula: Viability (%) =


$$\text{Viability (\%)}=\frac{OD570\;of\;treated\;cells}{OD570\;of\;control\;cells\;}\;\times \;100$$

### 2.10 Ex vivo hemolysis assay of the biosurfactant crude extract

The ex vivo hemolysis assay was conducted according to the method, with some modifications, described by Zhou et al. (2017). Briefly, freshly collected human red blood cells (RBCs) were centrifuged for 10 min at 2500 rpm, then washed three times and diluted to a final concentration of 5% v/v in sterile PBS (pH 7.4). Afterward, 500 µl of the diluted RBCs were added to 500 µl of crude biosurfactant extract at a final concentration of 0.5X MIC (0.78 mg/ml). Triton X-100 (0.1% in PBS), which can lyse RBCs completely, was used as the positive control while PBS buffer was used as the negative control. Shaking and incubation of samples at 37°C for 1 h was done and then samples were centrifuged again at 2500 rpm for 15 min. The supernatant was collected and transferred to wells of a microtiter plate, and its optical density was recorded by an ELISA plate reader at 545 nm. Sterile PBS (pH 7.4) was used as a blank. The same test was repeated with the addition of biosurfactant and DMSO-impregnated catheter to diluted RBCs. The hemolysis percentage was estimated using the following equation:


$$Hemolysis\;percentage\;\left(\%\right)\;=\;\frac{\;A_{s}\;-\;A_{n\;}}{\;A_{p}\;-\;A_{n}\;}\;\times \;100$$

where A_s_, A_n_, and A_p_ are the absorbances of the sample, negative, and positive controls, respectively. The assay was calculated as the mean ± standard deviation of three replicates.

### 2.11 Gas chromatography-mass spectrophotometry analysis

Five milligrams of the extract dry weight was mixed with 120 µl silylating agent (N,O- Bis (tert-butyl dimethyl silyl) acetamide and incubated at 60°C for 30 minutes and analyzed by gas chromatography. The GC apparatus (Shimadzu^®^, Kyoto, Japan) coupled with a QP2010 Rtx-5MS was used to identify the structural analog of the crude biosurfactant. Helium was employed as a carrier gas, and a total of 10 µl of the sample was added to the apparatus. The runtime was 45 minutes with a flow rate of 1.24 ml/min. The oven was maintained at a temperature ranging from 60 to 260°C. The data were processed by matching the mass spectra and retention indices of peaks with references to retention index and mass spectra from the National Institute of Standards and Technology (NIST) library.

### 2.12 Statistical analysis

Statistical analysis was performed using GraphPad Prism 8.0.0 for Windows (GraphPad Software Inc., CA, USA). Independent samples t-test and two-way ANOVA (analysis of variance) were employed to analyze the statistical differences between crude biosurfactant and DMSO-treated cultures, where *p*<0.05 was considered to be statistically significant.

## 3 Results

### 3.1 Screening for biosurfactant-producing endophytic bacteria

**Figure 1:**
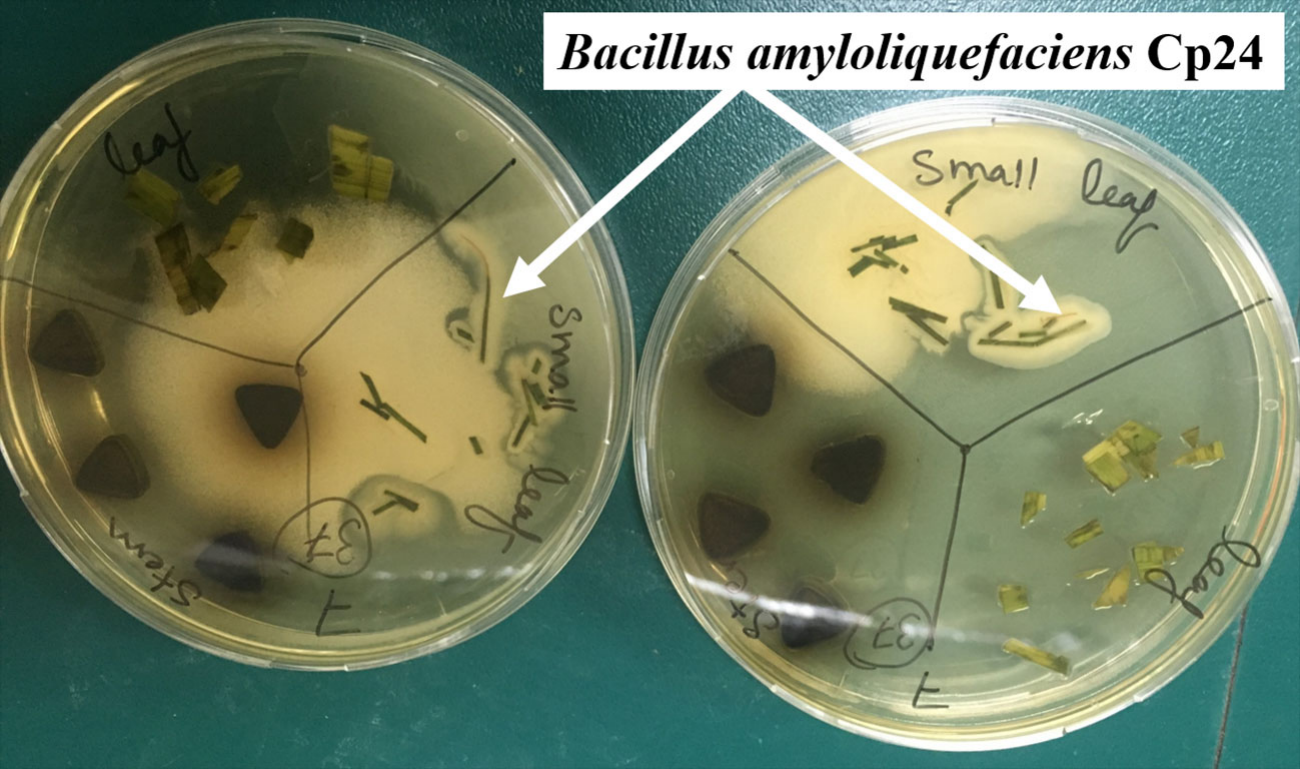
Growth of the endophytic isolates CP24 from the leaf fragments of *C. papyrus*.

In all, 10 plant samples were collected throughout the study period from different locations along the banks of the River Nile. A total of 44 bacterial endophytes were isolated from these plants. The plant species, their sites of collection, and the morphology of the isolates recovered from each plant are listed in **Supplementary Table 1**. CFSs of all endophytes were screened for their biosurfactant activity *via* different qualitative and quantitative assays. Of all endophytes that showed a positive drop collapse test, the CFS of an endophyte coded “Cp24” showed the highest biosurfactant activity. It produced a clear zone of 74 mm diameter in the oil dispersion assay and an %EI24 of 60 ± 2.5. Cp24 was isolated from the leaf of *Cyperus papyrus* collected from the Pharaonic Village in Cairo (29.9973° N, 31.2148° E). The growth of Cp24 from the leaf fragments of *C. papyrus* on TSA plates is shown in **Figure 1**.

### 3.2 Identification of endophytic isolate-producing biosurfactant

**Figure 2:**
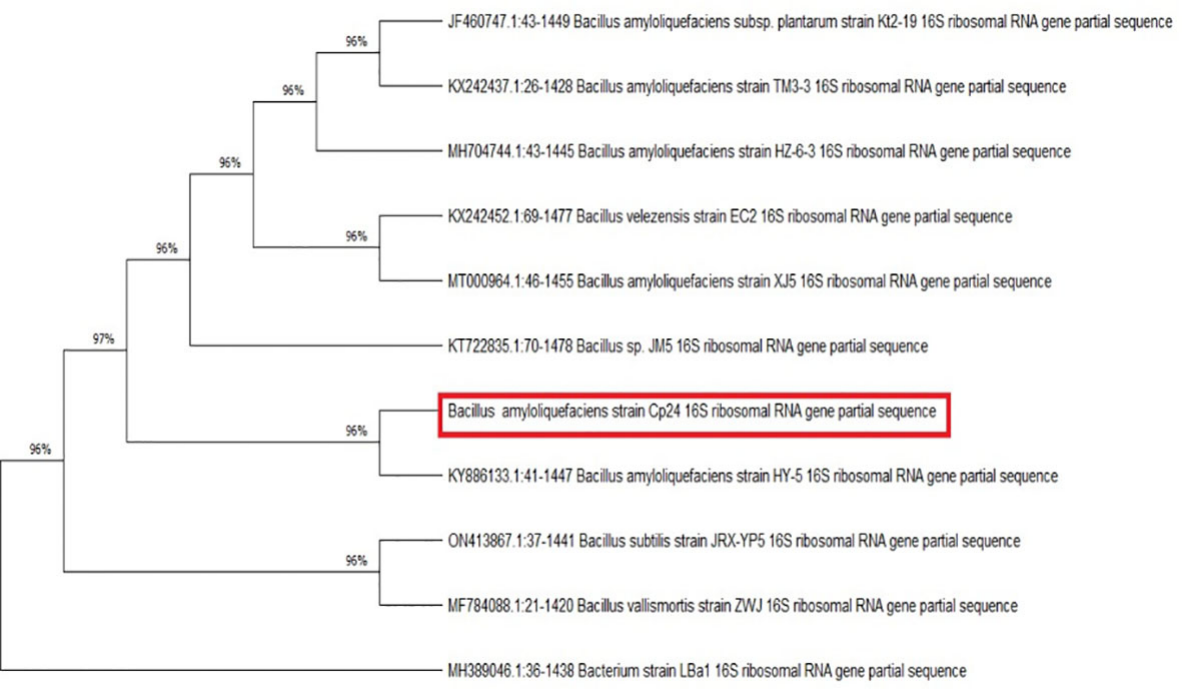
Evolutionary relationships between *B. amyloliquefaciens* Cp24 and highly similar strains. The phylogenetic tree was constructed by the maximum parsimony method (1000 replicates).

The endophytic isolate Cp24 grew on TSA plates as rough, opaque, fuzzy yellow to slightly orange colonies with jagged edges. Under a light microscope, it appeared as Gram-positive rods arranged in chains. The nucleotide sequence of the 16S *rRNA* gene carried by Cp24 showed the highest similarity to the corresponding gene of strain *B. amyloliquefaciens* HY-5 (GenBank accession: KY886133.1; percent similarity: 97.68%). A phylogenetic tree based on the 16S rRNA sequence of Cp24 and highly similar strains retrieved from the NCBI database showed that Cp24 was clustered with the same strain (**Figure 2**). Accordingly, Cp24 was tentatively identified as *B. amyloliquefaciens*.

### 3.3 Antibacterial activity of the crude biosurfactant against *A. baumannii* isolates

The crude biosurfactant extract of Cp24 (50 mg/ml) displayed considerable antibacterial activity against four MDR and XDR *A. baumannii* isolates in addition to the standard strain (ATCC^®^ 19606). The MIC values of the crude biosurfactant in the tested strains ranged from 0.78 to 1.56 mg/ml, as shown in **Table 2**.

**Table 2:** MICs of the crude biosurfactant produced by Cp24 against *A. baumannii* strains.

Bacterial Strain	MIC of Cp24 crude biosurfactant extract (mg/ml)
*A. baumannii* isolate M02	1.56
*A. baumannii* isolate M03	1.56
*A. baumannii* isolate M04	1.56
*A. baumannii* isolate M15	0.78
*A. baumannii* standard strain (ATCC^®^ 19606)	0.78

### 3.4 Sub-MIC concentration of crude biosurfactant extract significantly inhibits *A. baumannii* biofilm formation

**Figure 3:**
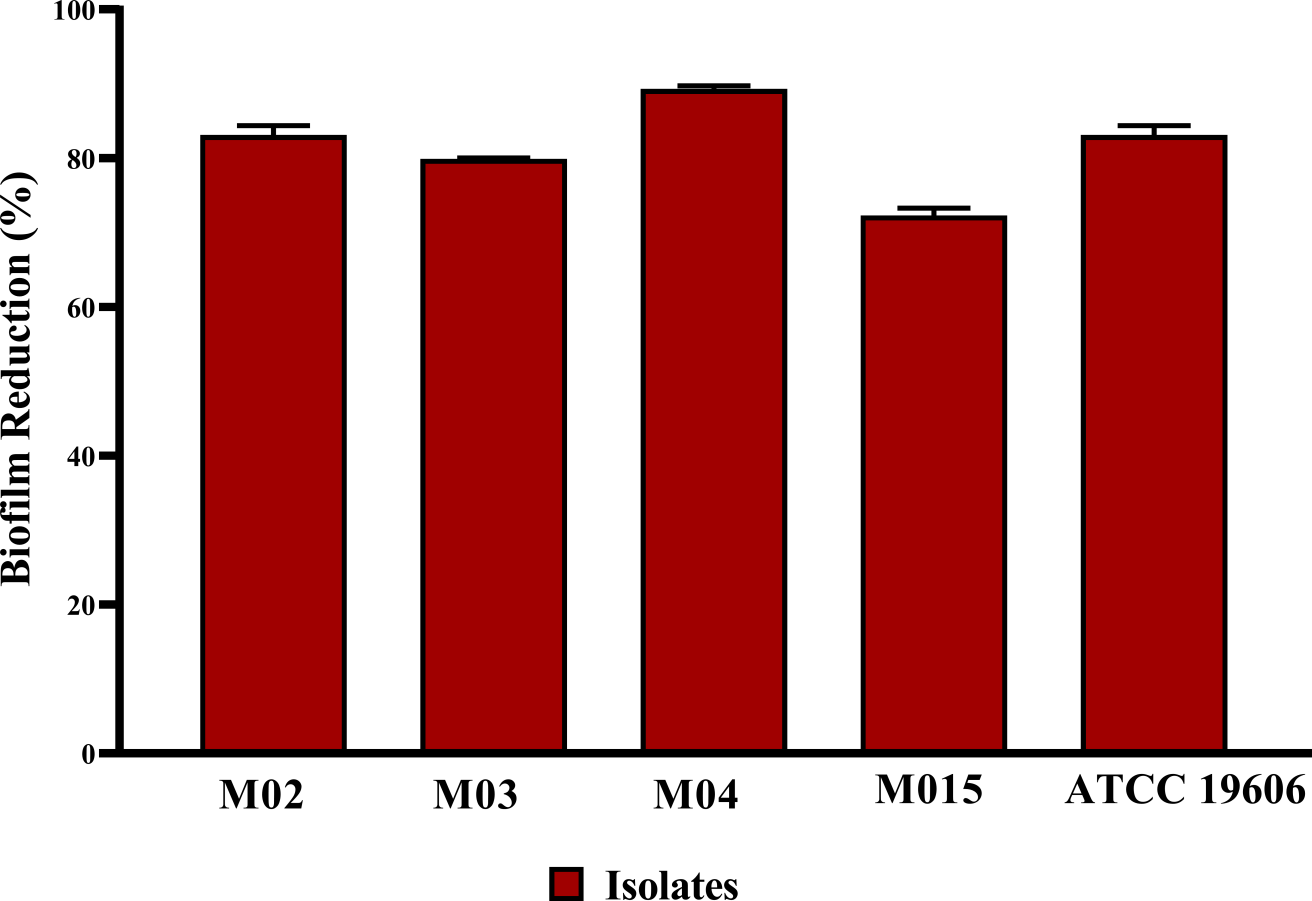
Quantification of the inhibitory effect of *B. amyloliquefaciens* crude biosurfactant on biofilms formation of *A. baumannii* isolates using crystal violet assay, showing the average reduction percentage of biofilm formation of *A. baumannii* isolates treated with 0.5X MIC of the crude biosurfactant Cp24 extract. Data represent the mean of at least three biological replicates, and error bars show the standard deviation.

All tested *A. baumannii* isolates and the standard strain displayed strong biofilm-forming capacity, with BFI ranging from 1.25 to 2.2. The antibiofilm potential of *B. amyloliquefaciens* Cp24 crude biosurfactant was determined by its ability to impair the biofilm formation of the tested strains and inhibit their adhesion ability to the surface without any effect on their growth. Our results showed that the *B. amyloliquefaciens* Cp24 crude biosurfactant efficiently inhibited biofilm formation of all the tested strains at half of its MIC. At this concentration, the biofilm formation among all the treated *A. baumannii* isolates was significantly inhibited (*p*-value<0.0001), with the percentage of inhibition ranging from 71.6 to 89.59% (**Figure 3**).

### 3.5 Eradication effect of biosurfactant crude extract on established biofilm of *A. baumannii*

**Figure 4:**
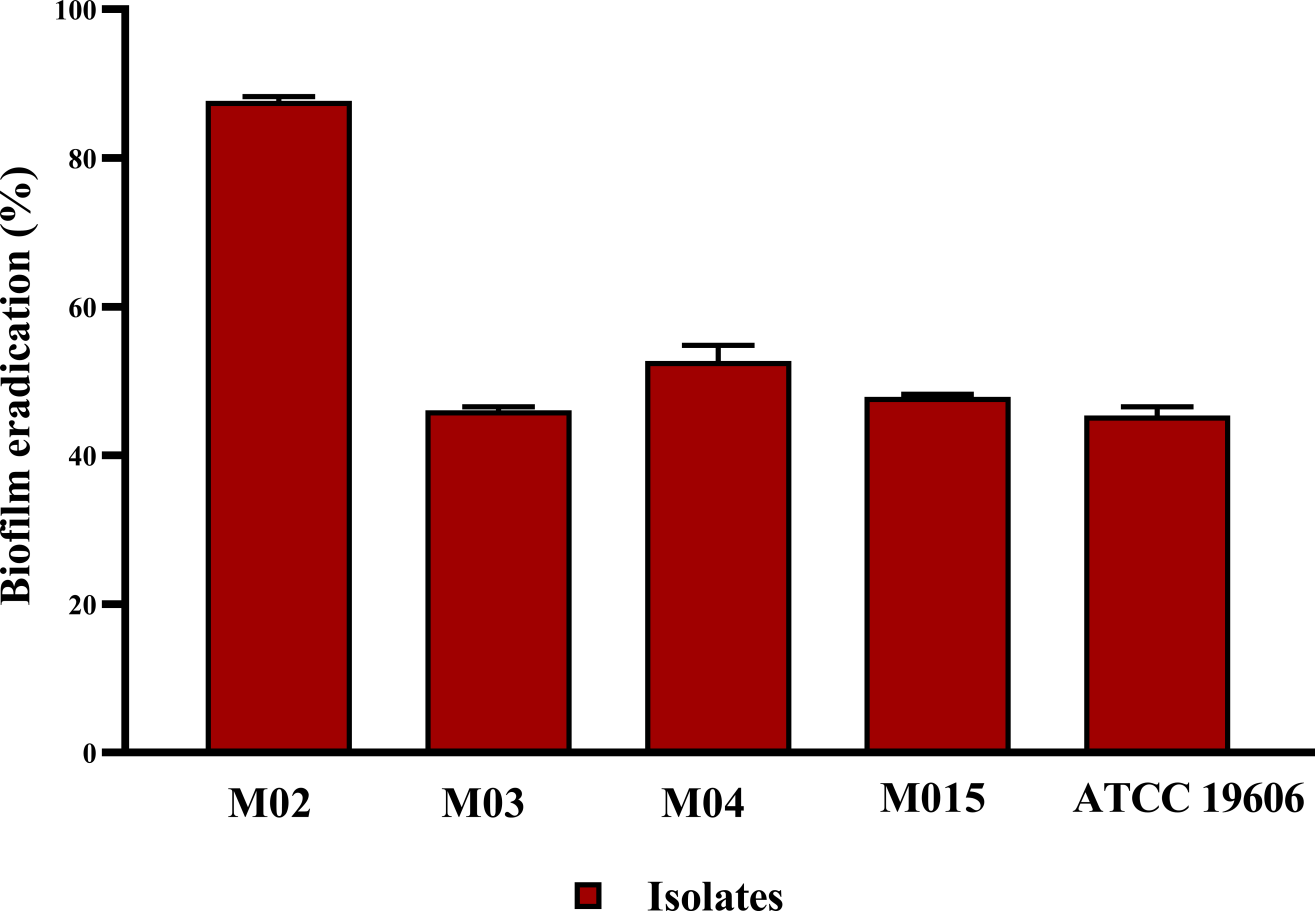
Quantification of the inhibitory effect of *B. amyloliquefaciens* crude biosurfactant on biofilms eradication of *A. baumannii* isolates using crystal violet assay, showing the average biofilm eradication percentage of established biofilms *A. baumannii* isolates. Data represent the mean of at least three biological replicates, and error bars show the standard deviation.

The antibiofilm potential of *B. amyloliquefaciens* Cp24 crude biosurfactant was further determined by its ability to impair the preformed biofilms of the tested strains. Our results showed that the *B. amyloliquefaciens* Cp24 crude biosurfactant efficiently disrupted the preformed biofilms. At the MIC concentrations, the eradication of the preformed biofilms by the *B. amyloliquefaciens* Cp24 crude biosurfactant ranged from 44.6% to 87.3% among all the tested strains (**Figure 4**).

### 3.6 *B. amyloliquefaciens* CVCs impregnated with crude biosurfactant have a significant *in vitro* antibiofilm activity against *A. baumannii*

**Figure 5:**
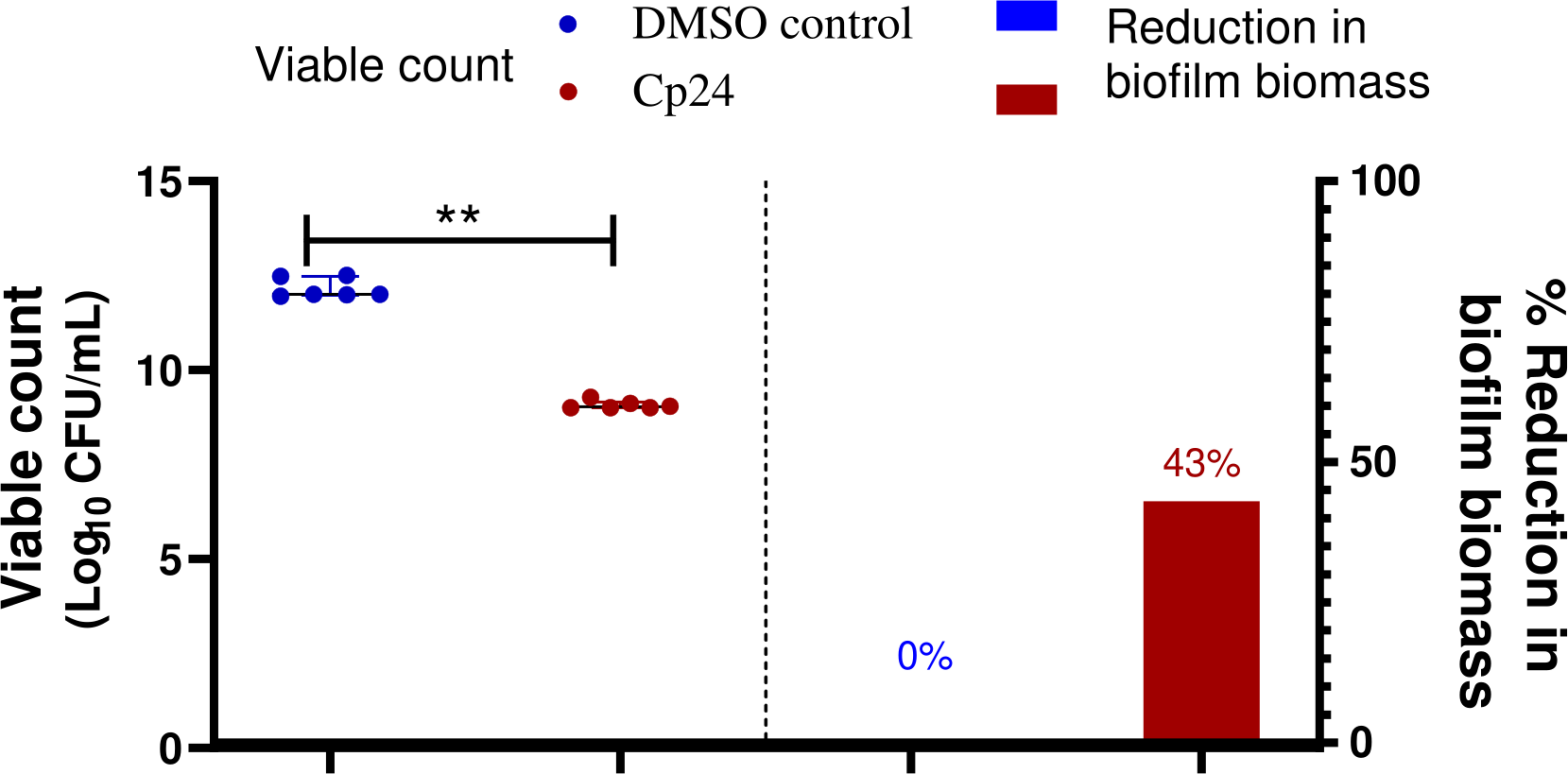
Quantification of the reduction in *A. baumannii* biofilm on crude biosurfactant extract (Cp24) impregnated CVCs compared to DMSO control. The percentage reduction in biofilm biomass was measured by crystal violet assay. The reduction in adhered viable bacterial counts of *A. baumannii* isolate M02 was measured by the drop plate method after 24 h of incubation. Data are represented as median with interquartile range and statistical difference was determined by student’s t-test, where statistical significance is represented by **p< 0.01.

Catheter impregnated with 0.5X MIC of the crude biosurfactant extract caused a significant reduction in the biofilm by three log_10_ cycles for the clinical isolate M02. The obtained results revealed that the viability of *A. baumannii* isolates M02 embedded in the biofilm were remarkably reduced upon coating by the Cp24 crude biosurfactant (**Figure 5**). The results obtained from the biofilm biomass quantification assay revealed that the impregnated CVCs caused a 43% reduction in biofilm formation.

### 3.7 SEM analysis revealed obvious antibiofilm activity of the impregnated CVC with the crude biosurfactant

**Figure 6:**
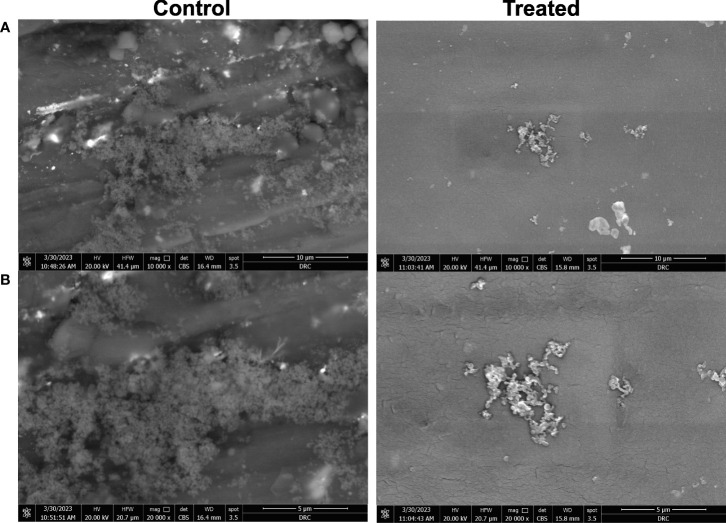
Microscopic visualization of biofilm formation by *A. baumannii* isolate M02 on CVC segments using scanning electron microscope (SEM). Biofilm formation on the catheter treated by impregnation with 0.5X MIC (0.78 mg/ml) of the crude biosurfactant extract (Cp24) was compared to the control DMSO-treated catheter. Visualization using SEM was done at magnifications of **(A)** 10,000 X and **(B)** 20,000 X.

The inhibition of biofilm formation on the impregnated CVC with the crude biosurfactant was confirmed by visualization using SEM analysis. *A. baumannii* growing on the control CVC surface showed higher bacterial cell density, while impregnated CVC showed scattered aggregation of fewer cells than the control (**Figure 6**).

### 3.8 Cytotoxicity assay for biosurfactant crude extract

**Figure 7:**
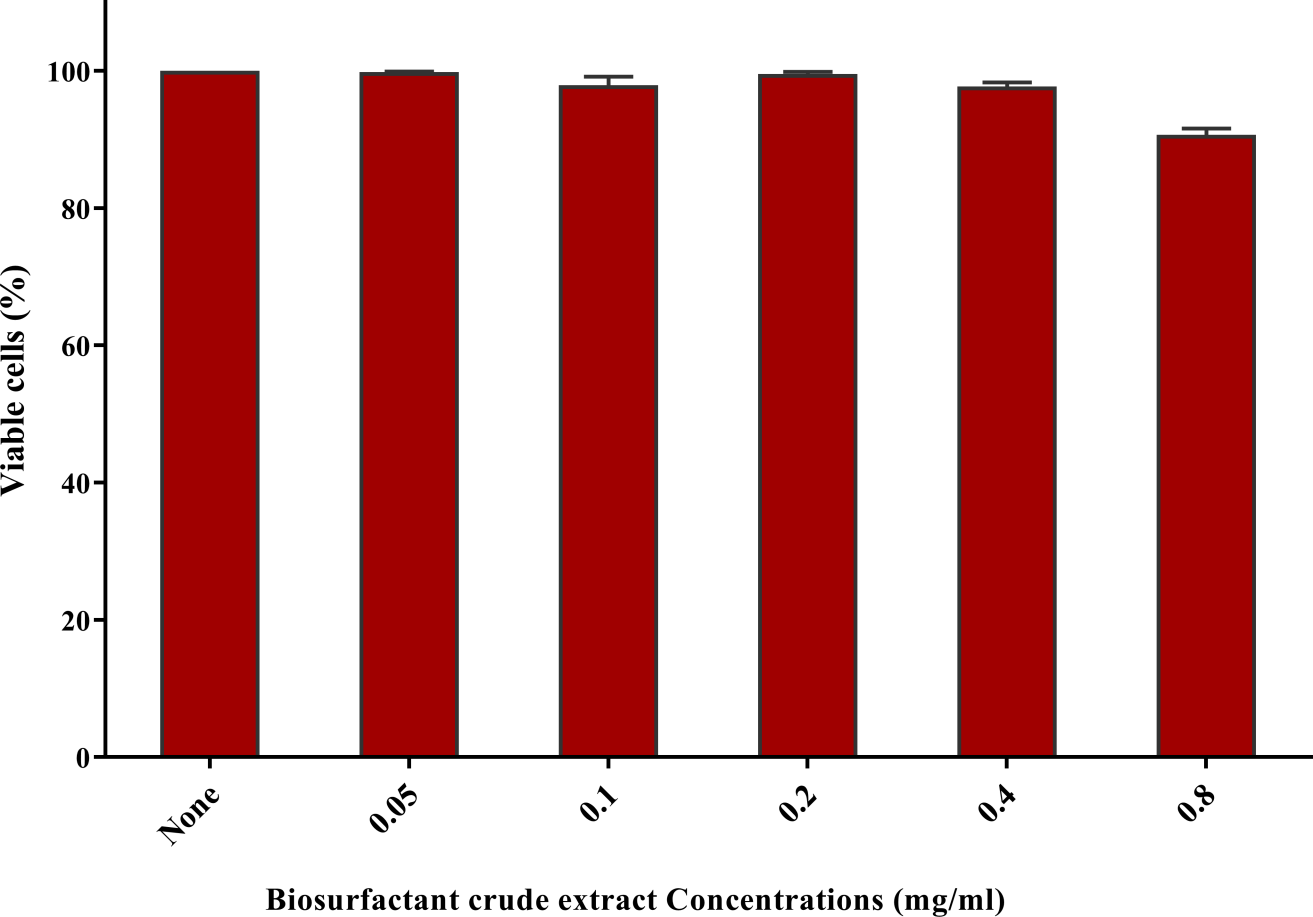
Cytotoxicity assay on human fibroblast cells after 24 h exposure to the nutrient medium interacting with serial dilutions of biosurfactant crude extract (0.05-0.8 mg/ml). Cytotoxicity was tested by sulforhodamine B (SRB) (0.4% w/v) and represented as the percentage of remaining viable cells after applying the treatment.

No substantial change was observed in the viability of skin fibroblast cells at sub-MIC concentrations of the crude biosurfactant extract when compared to the untreated control cells. The viability of fibroblast cells at the concentration used in catheter impregnation (0.78 mg/ml) was more than 90% (**Figure 7**).

### 3.9 Ex vivo hemolysis assay of the biosurfactant crude extract

The potential hemolytic activity induced by the crude biosurfactant of *B. amyloliquefaciens* Cp24 was investigated as an indication of ex vivo blood biocompatibility. The Triton X 100 was used as a positive control and turned red color due to hemolysis, resulting in the release of hemoglobin (Hb) from the red blood cells (RBCs), whereas the negative control (PBS solutions) did not show visible hemolysis. Crude biosurfactant at a concentration of 0.78 mg/ml showed hemolysis of 6.6%. However, impregnated catheter with an equivalent concentration (0.78 mg/ml) caused a lower hemolytic effect, of 2.3%.

### 3.10 Gas Chromatography-Mass Spectrometry (GC–MS) Analysis

**Figure 8:**
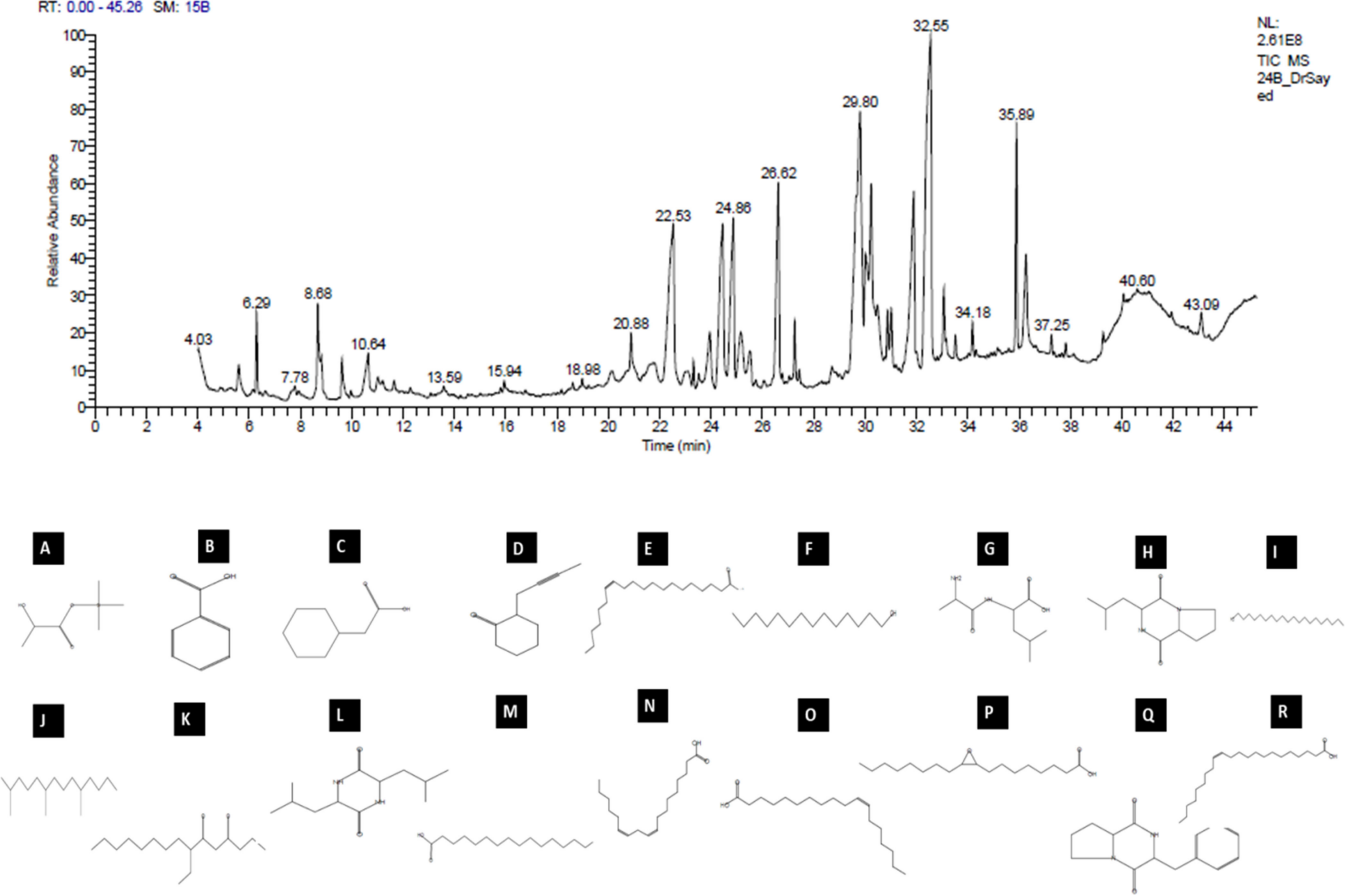
GC–MS analysis of the crude biosurfactants derived from *B*. *amyloliquefaciens* Cp24 endophyte. Identified compounds: **(A)** D-(-)-Lactic acid; **(B)** Benzoic acid **(C)** Benzeneacetic acid; **(D)** 2-(2-butynyl) Cyclohexanone; **(E)** cis-13-Eicosenoic acid; **(F)** 1-Tetradecanol; **(G)** dl-Alanyl-l-leucine; **(H)** [Cyclo (D-Leu-L-Pro)]; **(I)** 1-Eicosanol, **(J)** 2,6,10-Trimethyltetradecane (Farnesan); **(K)** 7-Ethyl-4,6-heptadecandione;**(L)** [Cyclo(Leu-Leu)]; **(M)** n-Hexadecanoic acid; **(N)** 9,12-Octadecadienoic acid; **(O)** cis-Vaccenic acid; **(P)** cis- (Epoxyoleic acid); **(Q)** 3-Benzyl-hexahydro-pyrrolo[1, 2-a]pyrazine-1,4-dione; **(R)** Erucic acid.

The analytical technique GC–MS consists of gas chromatography coupled with mass spectroscopy and it was used for the detection of various compounds present in the *B. amyloliquefaciens* Cp24 crude biosurfactant. The GC–MS chromatogram showed different peaks, indicating the presence of different compounds (**Figure 8**). Major peak compounds at the respective retention time were identified from the standard library compound, and are shown in **Table 3**.

**Table 3:** Major constituents of the crude biosurfactants of *B. amyloliquefaciens* Cp24 endophyte using GC–MS.

Compound no.	RT	% Area	Compound name	Molecular Formula	Class
**1**	6.29	1.43	D-(-)-Lactic acid	C_6_H_14_O_3_	Short-chain fatty acid
**2**	8.83	0.69	Benzoic acid	C_7_H_6_O_2_	Aromatic carboxylic acid
**3**	10.64	1.3	Benzeneacetic acid	C_8_H_8_O_2_	Organic Acid
**4**	11.65	0.24	2-(2-butynyl) Cyclohexanone	C_10_H_14_O	Cyclic ketone
**5**	18.98	0.28	cis-13-Eicosenoic acid	C_20_H_38_O_2_	Long-chain saturated fatty acid
**6**	20.88	0.76	1-Tetradecanol	C_14_H_30_O	Long-chain fatty alcohol
**7**	21.75	0.79	dl-Alanyl-l-leucine	C_9_H_18_N_2_O_3_	Dipeptide
**8**	22.53	5.51	Cyclo (D-Leu-L-Pro) 3-Isobutylhexahydropyrrolo [1,2-a]pyrazine-1,4-dione	C_11_H_18_N_2_O_2_	Cyclic dipeptide
**9**	23.31	0.46	1-Eicosanol	C_20_H_42_O	Long-chain fatty alcohol
**10**	23.52	0.31	2,6,10-Trimethyltetradecane (Farnesan)	C_17_H_36_	Acyclic farnesane sesquiterpenoids (Branched alkanes)
**11**	23.95	1.38	7-Ethyl-4,6-heptadecandione	C_19_H_36_O_2_	Fatty Acyl
**12**	25.5	0.95	Cyclo(Leu-Leu) 2,5-Piperazinedione, 3,6-bis(2-methylpropyl)-	C_12_H_22_N_2_O_2_	Cyclic dipeptide
**13**	26.62	5.52	n-Hexadecanoic acid	C_16_H_32_O_2_	Long-chain saturated fatty acids, surfactant
**14**	29.65	6.15	9,12-Octadecadienoic acid (alpha-Linoleic acid)	C_18_H_32_O_2_	Long-chain unsaturated fatty acid
**15**	29.8	10.6	cis-Vaccenic acid	C_18_H_34_O_2_	Long-chain monounsaturated fatty acid
**16**	30.01	1.79	Cyclo(Leu-Pro) Pyrrolo[1,2-a]pyrazine-1,4-dione,hexahydro-3-(2 methylpropyl)	C_11_H_18_N_2_O_2_	Cyclic dipeptide
**17**	31.02	1.44	Oxiraneoctanoic acid, 3-octyl-, cis- (Epoxyoleic acid)	C_18_H_34_O_3_	Epoxy fatty acid
**18**	32.56	19.53	3-Benzyl-hexahydro-pyrrolo[1, 2-a] pyrazine-1,4-dione	C_14_H_16_N_2_O_2_	Organonitrogen compound
**19**	36.27	2.44	Erucic acid	C_22_H_42_O_2_	Monounsaturated very long-chain fatty acid

## 4 Discussion

In the present study, we aimed to screen the antibiofilm activity of endophytes that produce biosurfactants against multidrug-resistant *A. baumannii* isolates through the collection of plant samples along the banks of the River Nile. From 10 collected plant samples, a total of 44 bacterial cultures were isolated and screened for biosurfactant activity. Endophytic isolate Cp24, isolated from *Cyperus papyrus*, showed strong and instant biosurfactant activity and was selected for further study. *Cyperus papyrus*, commonly named papyrus and used by Ancient Egyptians as a writing surface, belongs to the Cyperaceae family and is one of the plants that are widespread in the African subtropical and tropical wetlands (Mburu et al., 2015). Little scientific studies are available on this plant and its associated endophytes.

Endophytic isolate Cp24 was further identified using 16s rRNA sequencing and found to be *Bacillus amyloliquefaciens*. Endophytic *B. amyloliquefaciens* is a well-known endophyte that has been used for biological control against crop diseases and insect pests. It is found to be a safe microorganism with proven excellence in plant colonization (Liu et al., 2017). Previous studies showed the antimicrobial activity of *B. amyloliquefaciens* through the production of secondary metabolites, such as low-molecular-weight lipopeptides, polyenes, phospholipids, amino acids, nucleic acids, and polyketides, as well as antimicrobial proteins (Koumoutsi et al., 2004; Luo et al., 2022).

We tested the effect of the produced biosurfactant against *A.baumanni*, which is the causative agent for many serious infections, including endocarditis, meningitis, necrotizing fasciitis, sepsis, urinary tract infections, skin and/or soft tissue infections, and pneumonia. Its ability to survive is significantly increased by the creation of biofilms, which also renders them resistant to desiccation and antimicrobial treatment. Microbial adherence to biotic and abiotic surfaces such as catheters, ventilators, or even gloves promotes the spread of the infection from one individual to another (Vijayashree Priyadharsini et al., 2018). In the current study, the crude biosurfactant of *B. amyloliquefaciens* Cp24 showed considerable antibacterial effects against all the tested *A. baumannii* bacterial strains with MIC ranging from 0.78 to 1.56 mg/ml. Similarly, the antimicrobial activity of several biosurfactants has been previously reported (Gomaa, 2013; De Giani et al., 2021). Moreover, reports suggest that biosurfactants can prevent the growth of biofilms and microbial adherence without exhibiting antibacterial action (Tahmourespour et al., 2011; Hamza et al., 2017). The Cp24 crude biosurfactant was also tested for its antibiofilm activity against both biofilm formation and eradication of established biofilms. The antibiofilm activity against all *A. baumannii* isolates reached up to 71.6-89.59% at 0.5X MIC (*p*-value<0.01). Additionally, it successfully eradicated the preformed *A. baumannii* biofilms with a range of 44.6% to 87.3% among all the tested strains. Although the particular mechanisms underlying the antibiofilm activity of biosurfactants are not yet fully known, biosurfactants may impact the interactions between microorganisms and surfaces in several ways, including (1) modifying the surface’s physicochemical characteristics, such as surface energy, hydrophobicity, and surface charge, which lessens microbial adhesion (Giri et al., 2019); (2) downregulating the biofilm-related gene expression (Ohadi et al., 2020); (3) rendering the biofilms more soluble, which favors bacterial detachment (e Silva et al., 2017); and (4) decreasing biofilm formation through the interference with quorum sensing (Paraszkiewicz et al., 2021).

Numerous studies have shown that the previous coating of catheter surfaces with biosurfactants (surface conditioning) reduced microbial adhesion and colonization (Falagas and Makris, 2009; Janek et al., 2018). Therefore, our study also aimed to investigate the *in vitro* antibiofilm efficacy of CVC impregnated with a crude biosurfactant. Applying biosurfactants for catheter treatment reduced biofilm formation by 43%, whereas a previous study by Janek et al. (2018) showed a reduction in adherence by 70% for weak biofilm-forming *Escherichia coli*. The extracted biosurfactant reduced the number of viable cells adhering to CVCs by three log_10_ cycles, whereas previous studies on biosurfactant-coated catheters showed a reduction in the viable count by one log_10_ (Rodrigues et al., 2004). In addition to their ability to reduce biofilm formation, the crude biosurfactant exhibited, at its sub-MICs, no cytotoxic effect on human cell lines nor hemolytic activity against human blood, which makes it safe for use. The cytocompatibility and safety of biosurfactants have been reported in previous studies (Rodrigues, 2011; Gupta et al., 2017).

The GC-MS analysis of the Cp24 crude biosurfactant revealed the presence of 19 compounds. The nature of the majority of detected compounds was the fatty acids and peptides components of the lipopeptide biosurfactant, which conforms with the previously identified nature of *B. amyloliquefaciens* biosurfactants as a lipopeptide surfactant (Sarwar et al., 2018; Wu et al., 2022). The complex nature of biosurfactants and their mosaic distribution of polarity, as well as branched or circular structures, give them remarkable physical properties compared to synthetic surfactants. Therefore, biosurfactants, such as surfactin, with their cyclic nature and dynamic surface properties can combine densely at the interface, increasing their surface activity and providing them with properties that make them suitable for many potential applications (Otzen, 2017).

Cyclic peptides detected in the Cp24 extract have been detected as products by many *Bacillus* sp. and have been reported for many potential applications in the agricultural, pharmaceutical, and biotechnology industries due to their dynamic surface properties (Sarwar et al., 2018). Previous studies showed that the diketopiperazines (DKPs) cyclopeptides ((Cyclo(Leu-Pro) and Cyclo (Leu-Leu)), similar to those detected in our extract, inhibit quorum sensing mechanisms and biofilm formation by various microorganisms, such as soft rot-causing pathogen *Lelliottia amnigena* RCE and *P.aeruginosa* (Rashiya et al., 2021; Kachhadia et al., 2022). Additionally, the presence of nitrogen atoms in DKPs makes them physiologically more stable compared to their counterpart lactones (Almohaywi et al., 2019).

There are three primary FA groups produced by *Bacillus* species: branched-chain FAs, straight-chain FAs, and complex FA types, such as cyclic, hydroxyl, or epoxy FAs. The fatty acid (FA) composition of bacterial cells differs depending on the species, and, as an adaptation to environmental changes is crucial for bacterial growth, the FA composition of the cell’s membrane fluctuates according to the environment. The crude biosurfactant extract is composed of a mixture of fatty acids with a range of short-chain fatty acids (Lactic acid) to long-chain fatty acids, varying from C16 to C22 saturated (cis-13-eicosenoic acid and n-hexadecanoic acid) and unsaturated fatty acids (9,12-octadecadienoic acid, cis-vaccenic acid, and erucic acid). In addition, it also contained epoxy oleic acid. Previous studies showed that some *Bacillus* species are capable of producing epoxy FAs, which are FAs with one or two epoxy groups with antibacterial capabilities (Diomandé et al., 2015). Unsaturated fatty acids, such as cis-9-hexadecenoic acid and cis-9-tetradecenoic acid, were reported to inhibit the quorum sensing system and subsequently reduce the biofilm formation and suppress motility in *V. cholerae* and *A. baumannii* ATCC 17978 (Nicol et al., 2018). Previous studies revealed that fatty acids and their derivatives reduced the virulence characteristics of *Chromobacterium violaceum* (Santhakumari et al., 2017) and *Vibrio* Sp. (Pérez-López et al., 2018). Higher molecular-weight biosurfactants displayed higher emulsification activity, according to a study by Martins and Martins (2018).

The compound 3-Benzyl-hexahydro-pyrrolo[1, 2-a]pyrazine-1,4-dione represents 19% of the Cp24 extract component and it was previously identified in *Exiguobacterium indicum* SJ16 extract isolated from the rhizosphere of *Cyperus laevigatus.* It showed significant quorum sensing inhibition against the reference *Chromobacterium violaceum* CV026 strain and also inhibited the biofilm formation of *P. aeruginosa* (Singh et al., 2019).

## 5 Conclusion

In conclusion, this study demonstrates that impregnating catheters with crude biosurfactant of endophytic B. *amyloliquefaciens* strain Cp24 effectively reduces biofilm formation of *A. baumannii*, a multidrug-resistant bacterium that belongs to global clones and has a strong biofilm-forming capacity. An additional advantage is the safety of this compound on human cell lines and its reduced hemolysis activity. GC-MS analysis confirmed the production of lipopeptides with cyclic peptide moiety providing them with dynamic surface properties that increased their surface activity. In addition, the presence of long-chain and epoxy-type fatty acids was observed, which reduced virulence factors and exhibited quorum sensing inhibition activity, therefore reducing biofilm formation. Further studies should evaluate this approach by *in vivo* analysis via long-term catheterization in animal models and study the possible synergistic activity of these fatty acids with other antivirulence compounds to increase their activity.

## Data availability statement

The original contributions presented in the study are included in the article/**Supplementary Material**. Further inquiries can be directed to the corresponding author.

## Ethics statement

The study was performed in accordance with relevant guidelines and regulations and has been approved by the Ethics Committee of October University for Modern Sciences and Arts with the reference number M1/EC1/2023PD.

## Author contributions

All authors conceptualized the work, performed the experiments, analyzed the results, edited the manuscript, contributed to the article, and approved the submitted version.
