# Supplementary material for: Biosurfactant from Nile Papyrus endophyte with potential antibiofilm activity against global clones of Acinetobacter baumannii
Source: Front Cell Infect Microbiol. 2023 Jul 13;13:1210195. doi: 10.3389/fcimb.2023.1210195 (PMC10373939; doi:10.3389/fcimb.2023.1210195)
Supplement: Supplementary file 1 [file Table_1.docx]

**Supplementary Table 1: Plant species collected in the current study, their sites of collection, and the number and morphology of bacterial endophytes recovered from each plant**

| Location | Plant | No. of bacterial endophyte isolates | Morphology of bacterial endophytes |
| --- | --- | --- | --- |
| Zamalek | *Thevetia peruviana* | 4 | Gram-positive rods |
|  | *Water hyacinth* | 4 | Gram-positive rods |
| Pharaonic village | *Cyperus alternifolius* | 3 | Gram-positive rods |
|  | *Cyperus papyrus* | 6 | Gram-positive rods |
| Helwan | *Ipomoea palmata* | 1 | Gram-negative rods |
|  |  | 3 | Gram-positive rods |
|  | *Phragmites communis* | 2 | Gram-positive rods |
|  | *Ipomoea palmata* | 2 | Gram-positive rods |
| Meet Ghamr, Al-Mansoura | *Myriophyllum spicatum* | 1 | Gram-negative rods |
|  |  | 1 | Gram-positive rods |
|  | *Ipomoea fistulosa* | 3 | Gram-positive rods |
| Kasr Al-Ainy | Unidentifiable | 3 | Gram-positive rods |
|  | *Ficus decora* | 1 | Gram-positive rods |
|  | *Convolvulus* species | 5 | Gram-positive rods |
|  | *Plantago major* | 4 | Gram-positive rods |
|  |  | 1 | Gram-negative rods |
